# How to Work the Brain

**Published:** 1881-09

**Authors:** 


### HOW TO WORK THE BRAIN.

There are among our readers a large number of young people, many of them looking forward to or just entering upon an intellectual life, in some form—either in literature, the ministry, law, medicine, or some other field wherein their powers, both as students and thinkers, should be so developed and disciplined as to secure the greatest and best results.

The class of young men who imagine that there is some occult connection between gin and genius, and that late hours and a general Bohemian life are essential to literary and artistic advancement, is much smaller than twenty years ago. But there are many young fellows coming on who think that a certain carelessness of life and manner—an autocratic sort of independence—a grand contempt for conventionalities, and a superb scorn of regularity of all sorts—such as has characterized a few great geniuses in the world, is just the thing for them to affect.

Yet, if there is anything which even a very clever young man should congratulate himself on, it is the knowledge, early acquired, that he isn't a genius. For, if he thinks otherwise, the chances are a thousand to one that the mistake may spoil him, while if he prove to be a genius the world will be sure to find it out before he does.

Among the hackneyed definitions of genius none are better than these: "Genius is only great perseverance—patience—earnestness;" or, "Genius is only a great capacity for receiving discipline." There is a vast difference in natural gifts, but the "genius for doing fourteen hours of hard work per day" is the sort that has changed the face of the world.

It is of the first importance, therefore, to know how to work to advantage. And one of the best rules is to learn how to organize your time. That is what Lord Bacon meant in saying, "to choose time is to save time." And by that we have no idea that the wise man meant that we should choose to burn the midnight oil as a regular habit, nor that we should loaf around the edges of our work, as it were, waiting for "a mood" to come and tell us to take hold.

The habit of our most healthful, cheerful, lasting literary workers—those who do the most of the best kind of brain labor—is to apportion their time so as to bring all their duties within certain prescribed limits, and then to do each task in its own time—extraordinary events alone preventing. In this they are wiser than the ministers, for example, who form the vicious habit of delaying the preparation of their sermon until the last moment, and then drive it through with a rush. Much good work is done under a stress like this, but nature takes notice of it—that watchful and inexorable guardian, who knows neither pity nor injustice.—*Golden Rule.*

